# Supplementary material for: Tead1a Initiates Transcriptional Priming Through the TEAD1a/YAP‐Notch1‐Spi1/Cebpα Axis to Promote Neutrophil Fate
Source: Adv Sci (Weinh). 2025 Aug 28;12(41):e05441. doi: 10.1002/advs.202505441 (PMC12591127; doi:10.1002/advs.202505441)
Supplement: Supplementary file 4 — Supplementary Dataset [file ADVS-12-e05441-s004.zip › 3-1.zip!/3-1/02-1.cluster/cluster2/Enrich/ego_kegg.pdf]

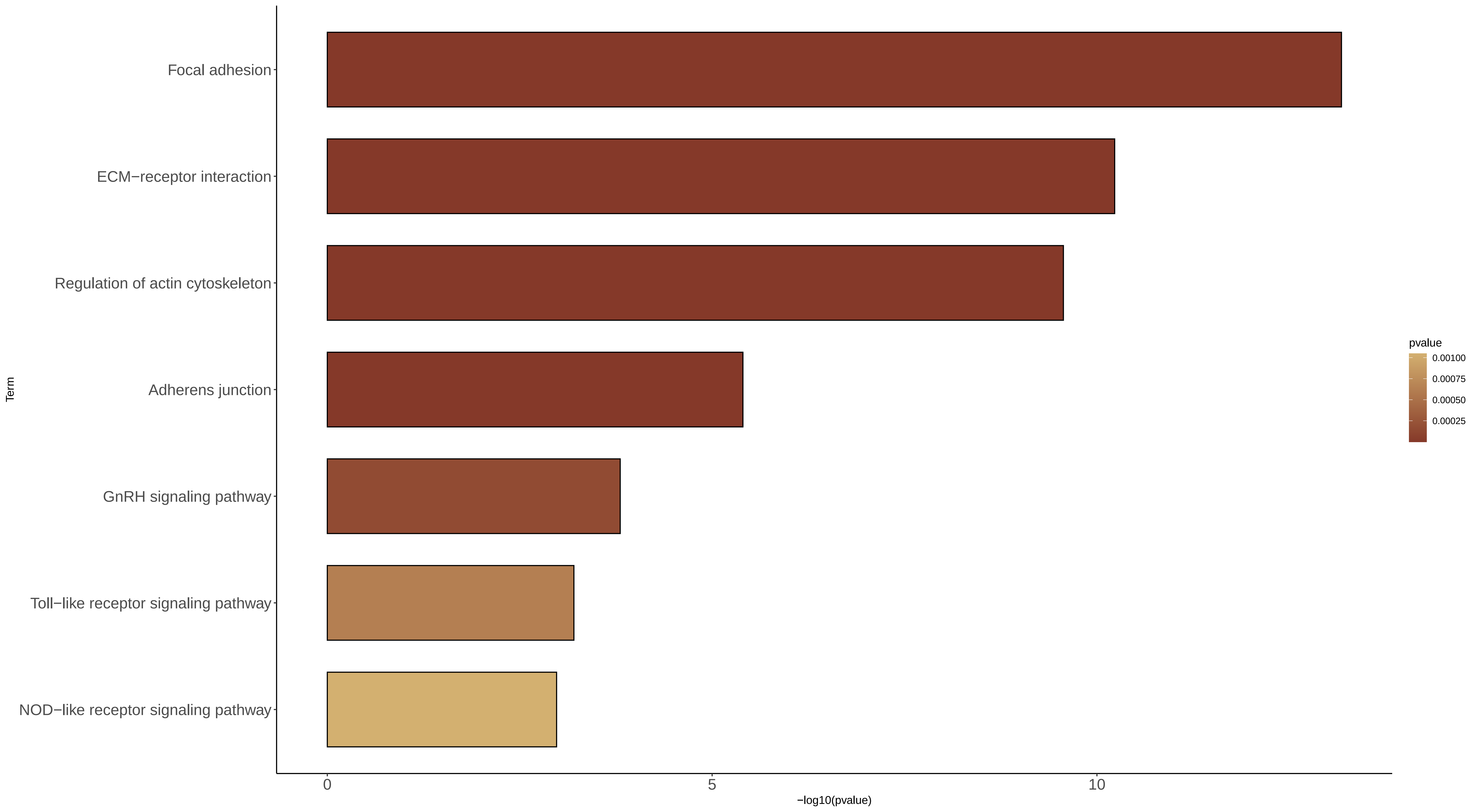

Focal adhesion
ECM−receptor interaction
Regulation of actin cytoskeleton
Adherens junction
GnRH signaling pathway
Toll−like receptor signaling pathway
NOD−like receptor signaling pathway
Term
0
5
10
−log10(pvalue)
pvalue
0.00100
0.00075
0.00050
0.00025
